# Transformative adaptation in times of polycrisis: insights from Milan’s New Urban Economies

**DOI:** 10.3389/fsoc.2026.1804091

**Published:** 2026-05-08

**Authors:** Cristiana Zara, Francesca Forno

**Affiliations:** Department of Sociology and Social Research, University of Trento, Trento, Italy

**Keywords:** hybrid organisations, Milan, New Urban Economies, sustainability transitions, transformative adaptation, urban polycrisis

## Abstract

European cities are increasingly shaped by polycrisis, where economic instability, social inequality, environmental stress, and institutional fragmentation intersect and reinforce one another. In this context, growth- and competitiveness-driven urban strategies often deepen exclusionary dynamics, even when framed as sustainable or innovative. At the same time, cities have seen the rise of New Urban Economies (NUEs) that seek to re-embed economic activity within social, ecological, and democratic values. The article examines how NUEs respond to these contradictory conditions through the lens of transformative adaptation, understood as a situated and political process of negotiating tensions between economic viability and socio-ecological commitments. Drawing on a typology of coping, incremental, and transformative adaptation, it argues that adaptation unfolds as a continuum of overlapping and hybrid strategies rather than a linear pathway to transformation. Empirically, the study focuses on Milan, a global innovation hub marked by strong sustainability narratives alongside acute housing pressures, inequality, and environmental stress. Based on a mixed qualitative methodology, including a georeferenced mapping of 87 NUEs and 25 in-depth interviews, the analysis shows that adaptive strategies are differentially distributed and shaped by sectoral conditions, institutional environments, and organisational trajectories, with initiatives combining and navigating multiple adaptive logics rather than following predefined pathways. Overall, the article conceptualises adaptation as a process of strategic positioning within urban polycrisis, through which urban economic actors negotiate constraints, sustain value-oriented practices, and rework the boundaries of transformation. In doing so, it contributes to urban sociology and sustainability transitions by understanding urban economic innovation as contested, situated, and continually reworked rather than predetermined.

## 1 Introduction

In recent years, European cities have become emblematic sites of polycrisis, where economic, social, environmental, and institutional crises overlap and reinforce one another, profoundly reshaping patterns of production, consumption, and urban life. In this context, urban development strategies centred on growth, competitiveness, and global attractiveness increasingly reveal their limitations, often exacerbating socio-spatial inequalities, environmental pressures, and processes of exclusion (Brenner and Theodore, 2002; Istituto Nazionale di Statistica (ISTAT), 2024). At the same time, cities have witnessed the proliferation of economic initiatives that seek to redefine the role of the economy itself, placing social, ecological, and democratic values at its core and experimenting with alternative ways of producing, distributing, and governing resources.

These initiatives, which we group under the label New Urban Economies (NUEs), encompass a heterogeneous array of entrepreneurial and collective practices operating at the intersection of social innovation, environmental sustainability, and economic transformation. These include social cooperatives, mission-oriented enterprises, solidarity-based purchasing groups (‘GAS’, in Italian), and hybrid community spaces that promote forms of economic activity that aim to re-embed production and exchange within collective needs, relational proximity, and ecological responsibility (Bruzzese, 2018; Magnaghi, 2020), thereby challenging extractive and profit-driven logics. In the literature on urban transitions and sustainability, NUEs have often been portrayed as laboratories for more inclusive and sustainable urban futures, endowed with transformative potential.

In this article, New Urban Economies are approached as an analytical category to identify a specific subset of urban economic initiatives emerging at the intersection of ecological and digital transitions. Building on the empirical criteria underpinning the mapping process, NUEs are defined as place-based, collectively organised economic activities that combine market-oriented practices with explicit social and/or environmental goals, often through hybrid organisational forms. While NUEs partially overlap with concepts such as social and solidarity economy and social innovation, they are not reducible to either. Unlike traditional social economy initiatives, NUEs include actors that operate within market and platform-based environments, engaging with processes of economic innovation beyond purely solidarity-driven logics. At the same time, they differ from broader notions of social innovation by focusing specifically on economic practices and organisational forms that are embedded in urban markets and territorial contexts. This delimitation does not aim to provide a definitive definition of NUEs, but rather to clarify their analytical scope within this study, where they serve as an empirical lens to examine how urban economic actors navigate and negotiate conditions of polycrisis.

Against this backdrop, the everyday operation of NUEs unfolds within increasingly adverse urban environments. Rising commercial rents, labour precarity, fragmented regulatory frameworks, and growing dependence on digital platforms place these initiatives under significant strain, calling into question their long-term viability and alignment with their founding values (Balducci et al., 2017; Meerow and Newell, 2019; Tozzi, 2023). Academic debates have frequently addressed these tensions through polarised lenses, either framing alternative economic initiatives as inherently fragile and marginal or depicting them as inevitably co-opted and normalised by dominant neoliberal market dynamics (Blythe et al., 2018; Feola, 2015).

This article positions itself critically with respect to dichotomous interpretations by proposing transformative adaptation as an analytical lens for examining New Urban Economies under conditions of polycrisis (Fedele et al., 2019). Rather than understanding adaptation as a purely functional adjustment or as a retreat from political ambition, transformative adaptation is conceptualised here as a situated, value-oriented, and intentional process through which initiatives seek to reconcile socio-ecological commitments with the material, institutional, and market constraints they face. From this perspective, adaptation constitutes a key arena of everyday negotiation, where tensions between economic viability and value coherence are not resolved once and for all, but are continuously reworked through organisational practices and strategic choices.

The article explores these dynamics through an empirical focus on Milan, a city that offers a particularly revealing context for investigating the interplay between innovation, crisis, and socio-economic transformation. As Italy’s economic capital and one of its most globalised urban centres, Milan combines dense innovation ecosystems, symbolic capital, and progressive policy agendas—especially in fields such as sustainability and food systems—with acute manifestations of socio-spatial inequality, housing pressure, rising costs of living and doing business, and environmental stress (Armondi and Bruzzese, 2017; Armondi et al., 2024). This combination makes Milan a fertile yet deeply contradictory terrain for New Urban Economies, where opportunities for experimentation and visibility coexist with structural constraints that disproportionately affect small, value-driven organisations.

By analysing how Milan-based NUEs navigate these conditions, the article sheds light on how transformative ambitions are not simply eroded by crisis or straightforwardly realised through radical rupture, but instead continuously negotiated, recalibrated, and, in some cases, reinforced through adaptive practices. In doing so, it contributes to urban sociology and sustainability transition studies by conceptualising urban economic innovation as an ongoing and situated process of transformation within crisis-ridden contexts and particular socio-spatial configurations shaped by urban inequalities, rather than as an outcome external to existing urban political and economic structures (Brenner and Theodore, 2002; Bruzzese, 2018).

Building on this perspective, this article makes three main contributions. First, it reconceptualises adaptation not as a merely technical or functional response to crisis, but as a situated and inherently political process through which urban economic actors continuously negotiate tensions between socio-ecological commitments and market constraints. Second, it challenges linear and teleological understandings of transformation by showing that adaptive trajectories do not unfold as a progression from coping to transformation, but rather as a dynamic continuum in which multiple adaptive logics coexist and interact. Third, drawing on empirical evidence from Milan’s New Urban Economies, the article advances the notion of hybrid and overlapping adaptation as a key analytical lens for understanding how transformative ambitions are sustained, recalibrated, or constrained in practice. In doing so, it contributes to ongoing debates by grounding urban economic transformation within the everyday socio-spatial practices, compromises, and power relations that characterise crisis-ridden urban contexts.

The article is organised as follows. First, a literature review establishes the theoretical framework of urban polycrisis and transformative adaptation. Second, the case of Milan and the research methodology are presented. Third, findings from the empirical analysis illustrate the spectrum of coping, incremental, and transformative adaptation. A discussion follows, deepening the interpretation of the findings. Finally, the conclusion synthesises the argument and its implications for urban studies and policy.

## 2 Situating adaptation in the context of urban polycrisis

In recent years, the notion of *polycrisis* has gained traction as a conceptual frame to capture the simultaneity, interdependence, and compounding effects of multiple systemic disruptions (Tooze, 2022; Rakowski et al., 2025). Rather than treating crises—economic, environmental, social, health-related, geopolitical—as discrete or sequential, the idea of polycrisis foregrounds their interactions, which often amplify instability, reduce institutional response capacity, and render urban futures increasingly uncertain. Urban contexts are particularly exposed to these dynamics. As Brenner (2019) and Harvey (2012) argue, cities function as key nodes within the architecture of crisis capitalism, absorbing and reflecting the contradictions of globalised accumulation, socio-spatial inequality, climate stress, and governance fragmentation.

### 2.1 Defining polycrisis

The concept of urban polycrisis is not merely descriptive; it serves an analytical purpose by revealing the compound and relational nature of crisis processes across scales. It links austerity to housing precarity, labour deregulation to infrastructural decay, and ecological degradation to institutional dysfunction. Crucially, polycrisis does not erupt as spectacular rupture but instead takes the form of chronic, layered strain embedded in the daily functioning of cities (Amin and Lancione, 2022; Simone, 2018). This calls into question technocratic approaches to resilience and demands a more grounded understanding of how crisis becomes entangled with the everyday production and governance of urban space.

For actors seeking to promote alternative urban economies, such as New Urban Economies, urban polycrisis constitutes both a barrier and a condition of possibility. On the one hand, escalating land costs, unaffordable housing, digital gentrification, and fragmented institutional support restrict access to space, resources, and political legitimacy (Armondi and Bruzzese, 2017; Peck and Theodore, 2015). On the other hand, the visible failure of dominant economic and urban models to address systemic vulnerabilities opens contingent spaces for experimentation, albeit under precarious and uncertain conditions.

The framing of crisis as polycrisis is also essential for interrogating the limits of prevailing urban policy and planning paradigms. While smart city agendas and green growth strategies often present themselves as adaptive responses to urban challenges, they tend to privilege techno-managerial fixes that obscure deeper socio-political inequalities (Clark, 2020; Datta and Odendaal, 2019; Montero, 2020; Swyngedouw, 2010). In contrast, viewing urban crisis as polycrisis invites an examination of how different actors—especially those operating at the margins—interpret, navigate, and respond to overlapping crisis conditions (Feola, 2015; Blythe et al., 2018).

This approach resonates with emerging scholarship on adaptation and urban transformation, which contends that responses to crisis are always mediated by power, positionality, and institutional context (Pelling et al., 2015; Patterson et al., 2017). Polycrisis not only shapes the types of adaptive strategies available to actors but also conditions their trajectories and transformative potential. It constrains, distorts, but also catalyses. Understanding adaptation in this context thus requires analytical attention to both the *structural conditions of crisis* and the *situated agency* of actors navigating them.

### 2.2 Typologies of adaptation and the situated complexity of urban transformation

Understanding adaptation in urban contexts requires moving beyond simplistic binaries between continuity and rupture. As socioecological crises become more intertwined and protracted, theorisations of adaptation demand a framework that captures both its temporal dimensions and its spatial embeddedness within the contradictions of contemporary urbanism. This section draws on Fedele et al. (2019) to outline a tripartite typology of adaptation—*coping*, *incremental*, and *transformative*—while situating these concepts within recent urban scholarship on adaptability (Andres and Kraftl, 2021; Andres, 2025). Adopting a critical stance towards transformative narratives, the typology is used here as a set of heuristic ideal types to interpret the diverse and often ambivalent adaptive practices of New Urban Economies, allowing for the identification of overlapping and hybrid configurations of adaptation within specific socio-spatial contexts.

Fedele et al. (2019) define *coping adaptation* as short-term, reactive responses aimed at managing immediate pressures without altering underlying structures or trajectories. These practices, while essential for survival, typically reinforce the status quo. *Incremental adaptation*, by contrast, involves more deliberate, stepwise adjustments to policy, governance, or practice, potentially leading to improved outcomes but remaining within existing systems and paradigms. Finally, *transformative adaptation* entails a deeper reconfiguration of structures, functions, values, and relationships to address the root causes of vulnerability, and is characterised by six key dimensions: restructuring, path-shifting, innovation, multiscalarity, system-wide change, and persistence. The threefold typology of adaptation is summarised in Table 1.

**Table 1:** Fedele et al.’s (2019) typology of adaptation.

Adaptation type	Core features	Temporal orientation	Relation to existing systems
Coping	Reactive, short-term responses aimed at maintaining continuity	Immediate/short-term	Reinforces existing structures
Incremental	Stepwise adjustments and innovations within existing frameworks	Medium-term	Works within dominant systems
Transformative	Structural reconfiguration of practices, relations, and institutions	Long-term	Challenges and reshapes existing systems

Drawing on relevant urban scholarship, it is important to emphasise that these categories should not be seen as rigid or mutually exclusive, but rather as part of a broader continuum of adaptive practice. Pelling et al. (2015) frame adaptation as a political and social process, involving contested decisions about what should be transformed, by whom, and for whose benefit. Patterson et al. (2017) highlight the role of governance dynamics—including power asymmetries, institutional capacities, and actor coalitions—in shaping both the scope and limits of transformation. Meerow and Newell (2019) stress that the values embedded in adaptation strategies often reflect prevailing political ideologies, potentially reinforcing existing inequalities. Feola (2015) critiques mainstream sustainability transitions for their neglect of political economy analysis, calling for deeper attention to structural barriers such as vested interests, institutional fragmentation, and path dependency.

These insights are particularly relevant when analysing urban economies in crisis. The neoliberal restructuring of cities has created a landscape marked by rising inequality, fragmented governance, and the privatisation of public goods (Brenner and Theodore, 2002; Harvey, 2012). Within this context, NUEs emerge as hybrid formations—often small-scale, community-rooted, and value-driven—that attempt to reconfigure urban economic life. However, not all NUEs operate as engines of transformation. Some function primarily within the logic of incremental adaptation, adjusting to shifting urban conditions without challenging systemic constraints. Others may exemplify coping responses, offering short-term resilience in the face of institutional neglect or economic precarity.

Importantly, even those initiatives that pursue transformative goals are embedded within contradictory urban conditions. As Blythe et al. (2018) warn, the discourse of transformation can itself obscure inequalities, overestimate agency, or depoliticise resistance. Efforts at transformation may be co-opted by incumbent actors, constrained by institutional inertia, or hindered by the sheer complexity of urban systems. O’Brien (2012) underscores the need to distinguish between *deliberate transformation*, driven by conscious intent and political vision, and transformation as a by-product of systemic disruption. In many cases, adaptive practices in urban contexts are better understood as processes of negotiation—between ambition and constraint, vision and survival, values and institutions.

This perspective is reinforced by recent urban theory on adaptability. Andres and Kraftl (2021) conceptualise adaptability through the lenses of *activation*, *trajectory*, and *responsiveness to context*, arguing that adaptation is always partial, relational, and embedded in temporality, as also highlighted in studies of urban alternatives in Milan (Bruzzese, 2018). Andres (2025) further develops this approach by examining ‘temporary urbanisms’ as both pragmatic responses to crisis and expressions of longer-term structural inequality. Accordingly, adaptation is not only about disruption or vision, but also about negotiation, compromise, and endurance—qualities often overlooked in dominant narratives—unfolding through practices that are socially and spatially embedded within uneven urban contexts.

The present study situates Milan’s NUEs within this conceptual frame. Rather than celebrating them as prefigurative utopias, the analysis views them as adaptive configurations that negotiate polycrisis through practices spanning the coping-transformative spectrum. Their significance lies not only in their ambitions but in how they confront constraints, manage trade-offs, and sustain value-driven orientations amidst crisis. In doing so, they offer insight into how transformation in urban settings is rarely heroic or totalising, but rather incremental, ambivalent, and persistently negotiated.

## 3 Case study and methods

### 3.1 Milan as a context of urban polycrisis and experimentation

Milan represents a particularly relevant case for analysing New Urban Economies under conditions of urban polycrisis, as it exemplifies key dynamics of contemporary neoliberal urbanism, where processes of globalisation, innovation-driven growth, and socio-spatial inequality are tightly intertwined. As Italy’s economic capital and a major European node of global connectivity, Milan concentrates advanced financial, creative, and knowledge-based sectors (Armondi and Bruzzese, 2017). Over the past two decades, the city has actively branded itself as a laboratory for innovation and sustainability through large-scale regeneration projects, international events such as Expo 2015, and policy agendas focused on smart-city and circular-economy transitions (Leonardi and Secchi, 2016). The city has increasingly adopted a sustainability and climate adaptation discourse, framed through flagship projects and international branding, which simultaneously promote urban competitiveness and real estate-led development (Tozzi, 2023). However, this trajectory has also produced phenomena of *ecogentrification*—where environmental improvements fuel displacement and exclusion of vulnerable groups—thus exposing the socio-political contradictions of green urbanism (Cucca, 2012).

Administratively, Milan is divided into nine *municipi*, each with delegated responsibilities, while remaining under strong centralised governance. This fine-grained structure corresponds to a highly uneven socio-economic geography, where central and semi-central areas generally exhibit lower social and material vulnerability compared to more peripheral zones, albeit with significant internal heterogeneity (Istituto Nazionale di Statistica (ISTAT), 2020). This landscape has been further reshaped by intense processes of regeneration and gentrification. Historically marginal neighbourhoods such as Isola, Lambrate, and Bovisa have transformed into hubs of cultural production and entrepreneurial experimentation (Armondi and Bruzzese, 2017; Semi, 2015). These transformations generate opportunities for innovative, sustainability-oriented initiatives while simultaneously intensifying real estate speculation, rising commercial rents, and displacement pressures. Consequently, Milan embodies a contradictory configuration where narratives of innovation and sustainability coexist with widening inequalities, labour precarity, and environmental stress (Brenner and Theodore, 2002; Tozzi, 2023).

Milan’s urban economy is shaped by the expansion of knowledge-intensive, creative, and digital sectors, including fashion, design, and platform-based services (Armondi et al., 2024; Mariotti et al., 2017). At the same time, the city has increasingly become a testing ground for proximity-based and relational economies, particularly in the aftermath of the COVID-19 pandemic, which renewed attention to neighbourhood-scale services, short supply chains, and temporary urban forms such as pop-up shops. While strategic planning instruments like the *Piano di Governo del Territorio* (PGT Milano, 2030) emphasise sustainability and neighbourhood regeneration, and initiatives such as the Milan Urban Food Policy Pact (MUFPP), launched after Expo 2015 and now expanded into a global framework for sustainable food systems, signal innovative urban policymaking, critical scholarship has underscored the ambivalent social effects of these policies and their entanglement with neoliberal urban restructuring (Balducci et al., 2017; Tozzi, 2023). It is within this context of overlapping crises and experimentation that Milan’s NUEs have emerged. Operating at the intersection of market activity, social reproduction, and ecological concern, they adopt hybrid organisational forms and rely on dense territorial networks while being deeply exposed to the competitive pressures of Milan’s urban environment. This makes Milan a revealing case for examining how transformative ambitions are negotiated and sustained in practice.

### 3.2 Research design and methodological approach

The study adopts a qualitative, multi-method research design combining spatial mapping with in-depth interviews and ethnographic fieldwork in order to capture both the systemic characteristics of Milan’s New Urban Economies and the situated practices through which initiatives confront conditions of urban polycrisis. This approach allows for an integrated analysis of macro-level urban patterns and micro-level organisational strategies. The research unfolded in three main phases: first, the construction of a georeferenced map of NUEs operating within the Municipality of Milan; second, the analytical refinement and classification of the mapped initiatives in line with an operational definition of NUEs; and third, the selection and qualitative investigation of a purposive sample of case studies through semi-structured interviews.

The first phase consisted of an extensive, iterative mapping of New Urban Economies. The aim was not to produce a closed or exhaustive inventory, but rather to develop a dynamic and analytically meaningful overview of urban value-oriented economic initiatives. The mapping process began with a preliminary ‘mapping of mappings’, drawing on a wide range of heterogeneous sources, including online directories and thematic maps produced by solidarity economy networks, environmental organisations such as Greenpeace, and civic platforms, as well as grey literature, previously published guides to sustainable and solidarity-based initiatives, and the researchers’ working knowledge of the local context.

This exploratory phase deliberately avoided strict sectoral boundaries in order to capture the diversity and hybridity of emerging urban economic practices. Initiatives were initially identified across a broad range of domains, including food, reuse and recycling, clothing and fashion, mobility, tourism, care, and hybrid or cross-sectoral activities. The mapping was subsequently deepened through targeted keyword searches aimed at strengthening sectoral coverage, particularly in Milan, where NUEs are particularly prominent in food, mobility, and digital innovation. Throughout this phase, the process was supported by iterative collective discussions within the research team and field-based activities, such as ethnographic urban walks. These moments served both to verify the existence and activity of initiatives and to better understand their embedding within neighbourhood-level social fabrics and urban regeneration processes.

The initial mapping produced a broad, heterogeneous list of initiatives, which required an analytical refinement phase to ensure coherence with the operational definition of New Urban Economies adopted. This ‘cleaning’ process involved a systematic, critical assessment of each initiative against a set of qualitative criteria. Initiatives lacking any market-oriented component, such as community gardens or any other self-provisioning activities, were not considered NUEs in a strict sense and were therefore reclassified as contextual actors within the wider urban eco-social landscape. Similarly, activities that were entirely public or that did not exhibit recognisable forms of organisational, social, or ecological innovation were excluded from the analytical sample.

**Figure 1:**
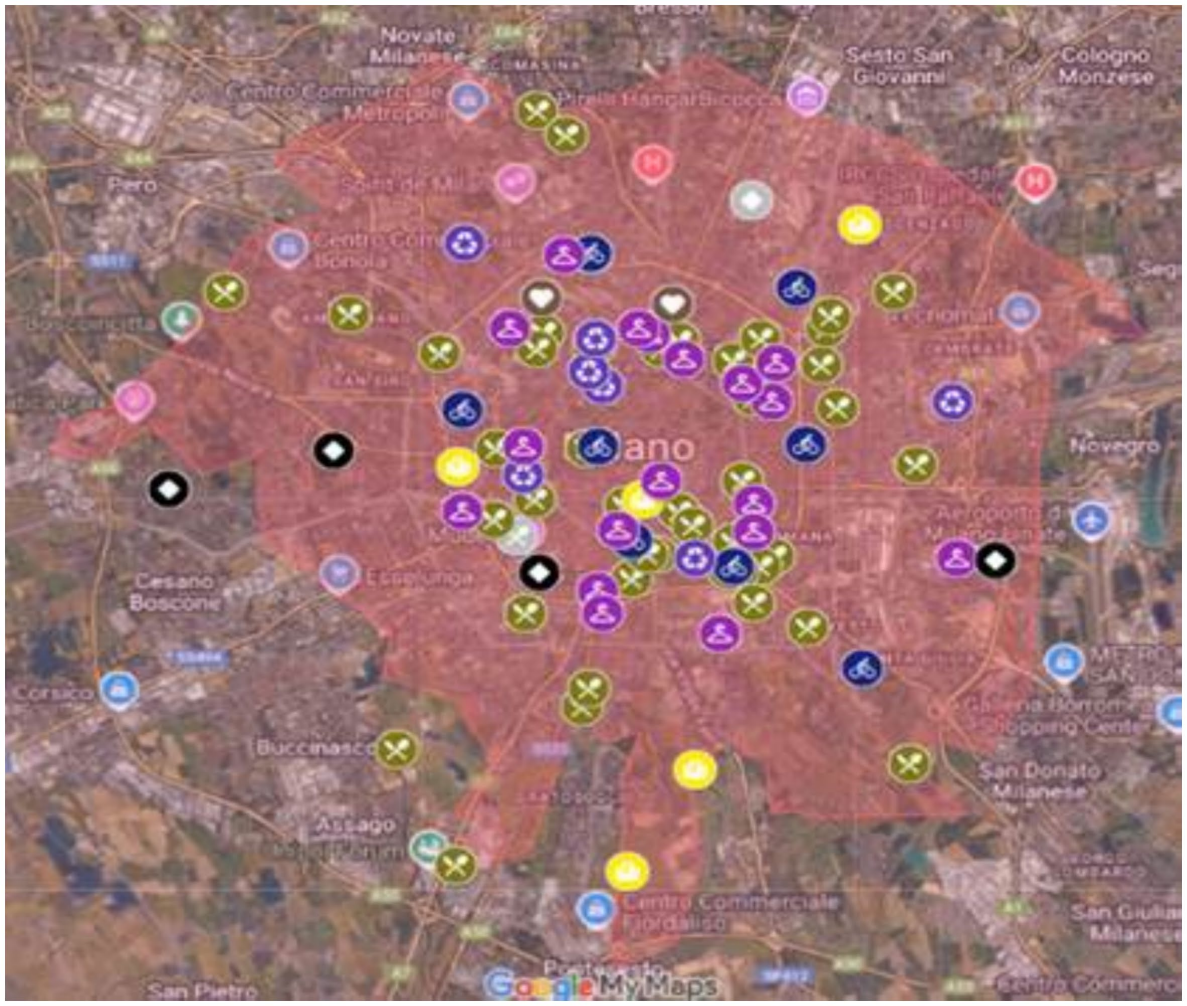
Detail of the map of NUEs in the Municipality of Milan.

Particular attention was also paid to the presence of a digital footprint or to the adoption of hybrid physical–digital models, which were treated as indicative of engagement with contemporary forms of urban economic practice. In addition, only initiatives that were demonstrably active and verifiable at the time of the research were retained. This refinement process resulted in the construction of a curated, georeferenced database of 87 NUEs finalised in May 2024. Figure 1 presents a preliminary mapping of NUE initiatives (March 2024), prior to the final refinement of the dataset.

The final dataset was classified into six main sectors—Food, Clothing, Reuse and Recycling, Mobility, Tourism, and Other—reflecting both empirical patterns and analytical priorities. This sectoral lens enabled a comparative analysis of how different regulatory environments, market structures, and spatial conditions shape the adaptive strategies and constraints faced by NUEs operating in distinct domains of urban life.

Primary qualitative data were collected through 25 semi-structured interviews, some of which involved multiple interlocutors, conducted between November 2024 and July 2025. Interviews had an average duration of over 1 h and explored a range of core themes, including the genesis of the initiative and its business model; founding values and internal tensions; relationships with territory, institutions, and formal and informal networks; perceived impacts of overlapping economic, social, and environmental crises; and future strategic orientations.

The selection of interviewees followed a purposive sampling strategy designed to reflect the diversity of Milan’s NUEs. The sample ensured coverage of all sectors included in the mapping, with a deliberate over-representation of food-related initiatives, reflecting both their numerical predominance and their strong connections to local policy frameworks such as the Milan Food Policy. Attention was also paid to operational diversity, including analogue, digital, and hybrid organisational models, as well as to temporal trajectories (founding period and longevity) and spatial distribution across central, semi-central, and peripheral neighbourhoods, in line with Milan’s differentiated socio-economic geography (Table 2).

**Table 2:** List of interviewed New Urban Economy initiatives in Milan.

Interview no.	Sector	Short description	Year of Foundation (YF)	Interview date	Interview duration
Int_1	Food	Neighbourhood-based food retail initiative focused on waste reduction, package-free and alternative consumption practices.	2021	17.03.2025	~1 h 20 min (not recorded)
Int_2	Food	Local urban farmers’ market encouraging consumption of local and seasonal food.	2018	21.03.2025	01:28:02
Int_3	Food	Grassroots food redistribution initiative addressing food waste and social vulnerability.	2015	24.03.2025	01:04:51
Int_4	Food	Independent food retailer emphasising short supply chains, packaging reduction and producer–consumer relationships.	2010	25.03.2025	01:20:14
Int_5	Food	Fast-food restaurant offering healthy, sustainable options, including vegetarian dishes.	2012	28.03.2025	01:28:52
Int_6	Food	Package-free food retail initiative with an educational mission.	2012	18.04.2025	01:16:22
Int_7	Food	Digital–physical platform supporting short food supply chains, package-free and producer–consumer coordination.	2013	15.05.2025	00:53:29
Int_8	Food	Network of farmers’ markets promoting local production and food sovereignty.	2015	28.05.2025	01:28:10
Int_9	Food	Plant-based and gluten-free restaurant.	2021	03.06.2025	~1 h 05 min (two interviews)
Int_10	Food	Solidarity-based purchasing group organised through collective and neighbourhood-level governance.	2000	06.06.2025	01:31:53
Int_11	Food	Informal consumer collective coordinating ethical food procurement at neighbourhood scale.	2008	09.06.2025	01:31:12
Int_12	Food	Hybrid urban space integrating food activities with cultural and social inclusion functions.	1991/2012 (project)	18.06.2025	01:37:45
Int_13	Food	Mission-oriented restaurant chain operating at scale while maintaining sustainability commitments.	2016	04.07.2025	01:09:39
Int_14	Mobility	Worker-led urban mobility initiative providing bike-based low-impact delivery services.	2013	10.01.2025	00:59:36
Int_15	Mobility	Bike-based social delivery platform combining digital coordination with ethical labour and sustainable mobility practices.	2020	05–10.02.2025	~1 h 40 min (two interviews)
Int_16	Tourism	Tourism initiative promoting responsible accommodation and sustainable urban tourism.	2015	23.05.2025	00:34:12
Int_17	Tourism	Rural–urban hospitality initiative linking agricultural activity with local tourism and education.	2016	30.05.2025	01:02:45
Int_18	Reuse/Recycling	Urban reuse initiative focused on waste reduction and circular economy practices.	2021	26.06.2025	01:14:46
Int_19	Clothing	Small-scale clothing initiative centred on sustainable materials and ethical production.	2022	26.11.2024	00:49:52
Int_20	Clothing	Independent fashion enterprise combining craftsmanship, sustainability, and local production.	2012	28.11.2024	00:57:09
Int_21	Clothing	Socially oriented clothing brand embedded in urban regeneration narratives.	2021	24.04.2025	01:19:28
Int_22	Clothing	Artisan fashion workshop operating within a regenerated urban neighbourhood.	2014	06.05.2025	00:52:02
Int_23	Clothing	Community-based clothing exchange and resale initiative promoting reuse and accessibility.	2009	14.05.2025	01:04:35
Int_24	Clothing	Large-scale second-hand clothing organisation linked to international solidarity networks.	1998/2006 (shop)	30.06.2025	01:01:00
Int_25	Other	Sustainable household cleaning products initiative developing plastic-free, water-soluble detergents.	2020	31.03–01.04.2025	~1 h 20 min (two interviews)

All interviews were transcribed and analysed using a combination of deductive and inductive coding. Deductively, the coding framework was informed by Fedele et al.’s (2019) typology of adaptation (coping, incremental, transformative), which provided an initial set of analytical categories. Inductively, the analysis remained open to emergent patterns, particularly with regard to the coexistence of multiple adaptive logics within the same initiative. Each case was therefore examined in relation to its dominant adaptive orientation, while also accounting for overlaps across categories. The classification was based on a qualitative assessment of organisational practices, strategic orientations, and narrated responses to urban constraints, as derived from interview material and contextual data from the mapping. This approach allowed for a flexible yet systematic categorisation, consistent with the understanding of adaptation as a situated and socio-spatially embedded process rather than a fixed attribute.

Crucially, a longitudinal dimension was integrated by analysing the foundation date of each initiative. These temporal trajectories proved essential for understanding the ‘DNA’ of NUEs, namely the socio-economic context and political opportunity structures at the time of their emergence, which continue to shape their adaptive capacities and constraints. This approach allowed the analysis to move beyond static spatial patterns and to examine how the geography and trajectories of Milan’s New Urban Economies intersect with longer-term processes of urban inequality and regeneration.

## 4 Reading Milan’s New Urban Economies through adaptation

This section provides an overview of Milan’s New Urban Economies by analysing their adaptive configurations through the lens of coping, incremental, and transformative adaptation. The aim is not to classify initiatives rigidly, but to map how different forms of adaptation emerge, overlap, and coexist in response to the constraints and pressures that characterise Milan’s contemporary urban environment. In this sense, as previously clarified, the typology of adaptation proposed by Fedele et al. (2019) is used as a heuristic device rather than as fixed, mutually exclusive categories.

### 4.1 Coping adaptation: sustaining alternatives under conditions of constraint

Coping adaptation characterises those New Urban Economies whose primary response to urban pressures consists of short-term and defensive strategies aimed at sustaining everyday operations and mitigating immediate forms of vulnerability. In the Milanese context, coping does not indicate passivity or lack of commitment. Rather, it reflects the need to operate under conditions of intense economic, social, and institutional constraint, where limited resources, unstable access to space, and fragmented policy support restrict the scope for longer-term strategic change.

Empirically, coping adaptation is particularly visible among initiatives that perform socially essential functions or that operate in sectors marked by low margins and high exposure to market volatility. These initiatives tend to prioritise continuity, relational work, and ethical coherence over growth or scalability. Their adaptive practices focus on buffering the effects of crisis—such as food insecurity, exclusion, or rising costs—without substantially altering the structural conditions that generate these problems.

A paradigmatic example of coping adaptation emerges from a grassroots food redistribution initiative that addresses food waste and urban poverty (Int_3). This NUE operates downstream in the agri-food system, redistributing surplus food to individuals and households experiencing economic fragility. Its activities respond directly to acute social needs that have intensified in recent years, particularly among elderly people, students, and precarious workers. While this initiative articulates a strong ethical critique of waste and inequality, its capacity to act remains closely tied to the availability of surplus, volunteer labour, and episodic institutional support. Engagement with public policies is described as necessary but difficult, often hindered by bureaucratic procedures that absorb time and energy without providing structural stability. This becomes particularly evident in the way the initiative describes the profiles of the people it reaches on a daily basis:

*“The people who benefit are people in vulnerable situations – unemployed people, elderly pensioners living in poverty, and also people involved in probation or alternative sentencing programmes. So yes, mostly people over 65, often older and living alone, in fragile conditions. Then there is also a whole group of students who have limited purchasing power, who get to know us and start coming because of that.” (Int_3: FOOD – Food redistribution, YF 2015)*

By focusing on elderly people living alone, students with limited purchasing power, and individuals involved in probation pathways, the extract makes visible the everyday forms of vulnerability addressed through food redistribution. The initiative’s adaptive response is oriented towards absorbing and mitigating these pressures as they emerge, rather than intervening upstream in the organisation of food provision or welfare. Here, transformative ambitions remain embedded in routine practices of care, shaped and constrained by the conditions of the urban environment in which it operates.

A case of a solidarity purchasing group rooted in neighbourhood-level collective organisation offers a different example of coping adaptation (Int_10). Unlike emergency-oriented initiatives, this case is not primarily reactive to crisis events; instead, it sustains an alternative mode of consumption centred on trust, ethical sourcing, and horizontal governance. Its adaptive strategy focuses on maintaining a stable community of participants and preserving relational infrastructures over time. However, this stability relies heavily on unpaid labour and personal commitment, resulting in ongoing challenges related to time availability, generational turnover, and the lack of supportive institutional frameworks.

These tensions become evident in how the initiative reflects on its relationship with local institutions and on the central role of voluntary labour in sustaining its activities:

*“There are no strong relationships with the local administrations. Sometimes you go to the Municipality with a proposal and they accept it, but there aren’t really any ongoing relationships. Partly because you don’t look for them, partly because you don’t need them, and partly because they don’t need you. When needs align, it’s easy to work together, but there are no stable ties.*

*(…)*

*At the same time, everything is based on volunteering. Without volunteers, GAS simply couldn’t stand on its own. But this also raises questions. How much can a GAS actually grow? Relying on volunteering also means taking work away from paid labour, and a small group of twenty people simply can’t afford to pay for all the activities involved. That’s why we started moving towards larger-scale solutions, like solidarity economy districts that brought together GAS groups, local producers, and social movements. But these experiences have been difficult to stabilise.” (Int_10: FOOD – Solidarity Purchasing Group, YF 2000)*

Here, coping adaptation takes the form of maintaining an enclave of alternative practice within a broader urban economy that offers few incentives for such models. The absence of stable relationships with local institutions and the reliance on voluntary labour are key factors enabling this NUE to sustain itself on a day-to-day basis. While the initiative embodies a clear normative critique of dominant market logics, its adaptive horizon remains largely defensive, focused on preserving an existing community and managing the limits of scale and labour recognition rather than expanding or institutionalising its practices.

Coping adaptation also characterises small-scale artisanal initiatives operating in highly competitive and weakly regulated sectors, such as fashion. Int_22 illustrates this form of individualised coping. As an artisan enterprise committed to quality, durability, and slow production, its main burden is to respond to rising costs, market saturation, and declining local demand by recalibrating expectations and combining restraint with pragmatic adjustments. These constraints are particularly evident in the way the initiative describes its production rhythms and its relationship with customers:

*“My approach is very much slow fashion. I don’t waste anything, I don’t have leftovers. Everything I produce, I sell. So, for example, with shirts, if a size is no longer available, the customer has to wait. They either wait until I make it specifically to order – which takes two or three weeks – or until the next round of production. I print the fabrics myself, maybe twenty metres at a time, and then I produce the shirts, the dresses, the skirts. And if something is finished, it’s finished.” (Int_22: CLOTHING – Artisan fashion, YF 2014)*

At the same time, sustaining such a model requires supplementing sales with additional income streams:

*“It’s really a moment where you have to vary a lot… I also do consultancy, I work for third parties… so diversifying production in order to keep the business economically sustainable.” (Int_22: CLOTHING – Artisan fashion, YF 2014)*

These excerpts illustrate how adaptive coping can manifest as resistance through restraint, coupled with economic diversification. Rather than pursuing growth, the initiative maintains a small-batch, low-waste production approach, while leveraging consultancy and commissioned projects to sustain slow fashion in a competitive urban market.

Across these cases, coping adaptation emerges as a necessary and widespread response to urban conditions that generate precarity and constrain experimentation. Whether oriented towards social redistribution, community-based consumption, or artisanal production, coping initiatives share a focus on maintaining alternative practices in the face of persistent pressure. At the same time, their experiences reveal the limits of coping as an adaptive strategy. While these initiatives provide essential social value and keep alternative imaginaries alive, their capacity to influence broader economic trajectories remains restricted. As the following sections show, other NUEs respond to similar constraints by pursuing incremental or transformative adaptations, sometimes in combination with coping practices, highlighting the diversity and hybridity of adaptive configurations within Milan’s New Urban Economies.

### 4.2 Incremental adaptation: innovating within dominant urban trajectories

Incremental adaptation accounts for a large share of Milan’s New Urban Economies and emerges, within this empirical sample, as the most common mode of response to urban pressures. Unlike coping initiatives, which prioritise continuity and mitigation, incremental initiatives actively innovate products, services, or organisational formats in order to remain competitive and economically viable. At the same time, these innovations largely unfold within existing market structures and policy frameworks, aligning with dominant urban trajectories rather than challenging them directly.

Empirically, incremental adaptation is marked by an explicit orientation towards stabilisation and, in many cases, controlled growth. Initiatives operating in this mode tend to frame sustainability as a value compatible with market expansion, scalability, and professionalisation. Innovation is often anticipatory rather than reactive, aimed at intercepting emerging consumer demands for ethical, healthy, or environmentally responsible products while preserving economic performance. This orientation resonates with Milan’s positioning as a hub for sustainable innovation, where selective policy support, visibility, and market opportunities favour initiatives that translate values into recognisable, replicable formats.

A paradigmatic example of incremental adaptation in the food sector is a ‘healthy fast food’ format that makes sustainable consumption accessible without sacrificing convenience or affordability (Int_5). Rather than questioning the organisation of the food system as a whole, the initiative focuses on product innovation, speed, and replicability, adapting sustainability to the temporal and spatial rhythms of urban consumption. Growth and expansion are explicitly framed as indicators of success, with innovation understood primarily in terms of format and efficiency. This orientation is reflected in the initiative’s ability to embed its model within large institutional settings:

*“We got in without a formal tender. They saw our format and liked it. People from Unicredit were like, ‘Oh, that’s nice!’. It wasn’t easy; we really had to push, but since 2018, we’ve been there, and they’ve kept us. Their canteen works through tenders and changes provider regularly, whereas with us, they chose continuity.” (Int_5: FOOD – Sustainable fast-food, YF 2012)*

The extract highlights how incremental adaptation operates through institutional recognition and format compatibility, allowing the initiative to secure stability by aligning sustainability with the routines and expectations of corporate environments.

A similar logic characterises a plant-based restaurant that combines ethical commitments with a strong focus on branding, design, and customer experience (Int_9). Here, sustainability is embedded in a lifestyle-oriented offering targeting environmentally conscious urban consumers. Incremental adaptation takes the form of qualitative growth, brand consolidation, and selective alignment with prevailing ‘green’ consumption trends, rather than systemic intervention in food provisioning. This orientation is grounded in sustained investment in product development and innovation. The following excerpt illustrates how this adaptive logic operates through continuous research and refinement, translating sustainability into technically sophisticated and market-compatible products:

*“There is a lot of research behind what we do. The shift to a 100% gluten-free menu alone required significant investment, time, and ongoing testing. Gluten-free products are almost never vegan, so developing fresh pasta that was both gluten-free and vegan was not easy. The same applies to leavened products and plant-based alternatives to cheese. It’s a highly innovative form of cooking, and also a very complex one.” (Int_9: FOOD – Plant-based restaurant, YF 2021)*

Incremental adaptation is not confined to the food sector. In tourism, a case of sustainable enterprise exemplifies a further register through which incremental strategies unfold (Int_16). While the above-mentioned cases of sustainable fast-food (Int_5) and plant-based restaurants (Int_9) foreground adaptation through product formats and investment in research and development, the tourist-based example illustrates how incremental adaptation can be articulated primarily through scale, finance, and investor confidence in highly competitive urban markets. The initiative positions itself as sustainable by promoting standards of “responsible” and “sustainable” short-term accommodation, mobilising sustainability as a marker of quality, professionalism, and regulatory credibility. Its trajectory originates in the context of the rapid expansion of short-term rentals in Milan:

*“2015 was the year of the Expo, and especially in Milan a major opportunity emerged around a market that was still very new in Italy at the time: short-term rentals.” (Int_16: TOURISM – Hospitality, YF 2015)*

Its subsequent development highlights a growth-oriented and financialised understanding of adaptation:

*“We are still one of the very few listed companies in the sector, with more than 500 shareholders. The company has grown very quickly, and people who invested with us in 2016 saw returns of twenty to twenty-five times their initial investment by 2019 – not too bad! What matters is that most shareholders are still there, very few have sold their shares, because the company’s growth continues to be central for them.” (Int_16: TOURISM – Hospitality, YF 2015)*

Here, incremental adaptation is enacted through the consolidation of scale and investor trust, demonstrating how sustainability-oriented narratives can coexist seamlessly with growth-driven, financialised logics in sectors deeply implicated in contemporary urban tensions.

By contrast, in sectors marked by intense global competition and limited policy support, such as fashion or household consumption, incremental adaptation often takes the form of niche positioning. Initiatives like the zero-waste fashion enterprise (Int_19) or the package-free shop (Int_25) interviewed promote sustainable alternatives through product differentiation, design-led strategies, and consumer education. Here, adaptation operates through the logic of ‘voting with one’s wallet’, relying on individual consumption choices rather than collective or institutional transformation.

In Int_19, the fashion and eco-design initiative producing accessories and garments articulates sustainability through an explicit commitment to zero-waste principles, understood as a design choice and a market proposition rather than as a strategy of economic containment:

*“Everything we do is driven by this commitment to zero waste. We work with small batches and only reproduce when it’s really necessary.” (Int_19: CLOTHING – Zero waste, YF 2022)*

This positioning results in a highly selective market niche, as reflected in the profile of its clientele:

*“It’s obviously not a mass-market product, because it requires a certain purchasing power. What we’ve noticed quite clearly is that many of our customers are connected to the design or creative industries. Often, they turn out to be journalists working on sustainability, or designers and architects – people who tend to have a stronger aesthetic and value-oriented sensibility than the average consumer.” (Int_19: CLOTHING – Zero waste, YF 2022)*

Taken together, these elements show how incremental adaptation in sectors such as fashion operates through ethically and aesthetically distinctive niches, whose reach remains closely tied to cultural capital and purchasing power.

Across the cases illustrated in this section, incremental adaptation appears as a pragmatic and often successful response to the constraints of Milan’s urban economy. By aligning ethical and environmental values with market-compatible strategies, incremental NUEs secure visibility, stability, and, in some cases, expansion. Yet, this mode of adaptation also reveals clear limits. By operating largely within existing economic and institutional arrangements, incremental initiatives tend to leave untouched the structural drivers of inequality, spatial exclusion, and environmental pressure embedded in urban life. As the following section shows, some initiatives aim to move beyond these limits by pursuing more explicitly transformative forms of adaptation, while others combine incremental strategies with broader systemic ambitions.

### 4.3 Transformative adaptation: reconfiguring economic relations, governance, and urban infrastructures

A smaller but analytically significant subset of Milan’s New Urban Economies pursues transformative forms of adaptation. Unlike coping and incremental configurations, initiatives operating in this mode do not limit their response to mitigating pressures or optimising existing models. Instead, they seek to rework the underlying relations through which economic activity is organised, engaging with questions of governance, access to space, labour, and the redistribution of value. Transformation here is not conceived as rupture or scale-driven growth, but as a gradual and contested process embedded in everyday organisational practices.

Empirically, transformative adaptation is marked by efforts to intervene upstream in the production of urban vulnerability. These initiatives articulate explicit ambitions to reshape power relations, institutional arrangements, and socio-material infrastructures, often positioning themselves as collective actors rather than as individual enterprises. Their adaptive strategies tend to combine economic activity with civic engagement, education, and political negotiation, blurring the boundaries between entrepreneurship, activism, and urban governance.

An exemplary case of transformative adaptation is represented by a multifunctional enterprise (Int_12), a long-term project of participatory urban regeneration centred on the collective management of a historic rural complex within the city. More than a venue for commercial activities, this NUE operates as a civic infrastructure in which economic functions are explicitly subordinated to social and cultural objectives. Its transformative capacity lies in the reconfiguration of urban space through a governance model that integrates economic sustainability, public access, and value-driven curation, embedding alternative values in the everyday use of space:

*“Our model is based on what we call ‘inhabitants’: services that occupy the spaces on a permanent basis. They pay rent but also actively participate in the project. It’s not a standard rental model – there is a rigorous process of selection and curation, both in terms of content and identity, and of values, with particular attention to the social dimension. We are managed by a social enterprise association that holds the concession from the Municipality of Milan. The inhabitants are an important source of income, but above all they are part of the project’s DNA, working with us to create a public programme of activities and services for the city.” (Int_12: FOOD – Multifunctional enterprise, YF 2012)*

Transformative adaptation also emerges in sectors such as urban logistics and mobility, where regulatory frameworks and infrastructural conditions strongly shape economic practices. A bike-based logistics case offers a clear illustration of this trajectory (Int_15). Unlike many delivery initiatives that emerge directly from courier-led experiences, this one frames logistics as a site of social and political intervention. From the outset, the initiative has combined low-impact delivery services with a strong emphasis on labour protection, safety, and the integration of social and solidarity-oriented activities. In doing so, it positions itself not simply as a service provider but as an actor seeking to reshape the norms governing urban logistics and broader access to the city. This orientation extends beyond organisational practices and translates into active engagement with public policy and grassroots mobilisation around urban mobility and the use of public space:

*“Public policy is really a key issue for us. This sector has a lot of visibility right now, and there’s also a lot of mobilisation from the ground up. There are associations and civic groups organising collective actions, sit-ins, and campaigns to demand more attention to public space, active mobility, people, and the environment. Milan is still a very congested city, with weak regulation and fragmented, often unsafe cycling infrastructure. So, voices are coming together from different directions – more grassroots, more organized – and they’re getting stronger. We really hope that, at some point, someone will actually listen to us.” (Int_15: MOBILITY – Bike-based logistics, YF 2020)*

In the food sector, a local farmers’ market represents a further expression of transformative adaptation through the reorganisation of agri-food relations (Int_8). Structured as a horizontally governed network of producers, the initiative prioritises direct relationships between producers and consumers, collective market management, and strong educational components. Its transformative ambition lies in challenging intermediary-dominated food systems and in re-embedding food provisioning within territorial, social, and ecological relations. Engagement with public authorities is often ambivalent and negotiated, reflecting tensions between the desire for autonomy and the need for access to urban spaces and regulatory recognition.

Transformative adaptation is also evident in initiatives that explicitly combine economic activity with educational and civic engagement. A package-free shop (Int_6) exemplifies this trajectory by integrating package-free retail with structured forms of environmental education, collaboration with schools, and engagement with local authorities and active citizens:

*“Our activity was created precisely for this reason: to bring together the sale of high-quality products and package-free retail with culture. The people who work in our shops are very well trained and highly prepared, because our goal is to carry out a form of drop-by-drop dissemination.” (Int_6: FOOD – Package-free shop, YF 2012)*

Here, transformation is pursued less through the scale of commercial operations than through the diffusion of cultural competences and participatory practices oriented towards a circular and collectively shared economy. By positioning itself as a node within a broader ecosystem of change—rather than as a standalone retail format—this NUE seeks to reshape everyday consumption while simultaneously strengthening civic capacity and institutional awareness.

Transformation, in these initiatives, does not occur without constraint. NUEs pursuing transformative adaptation face persistent economic pressures, spatial insecurity, and institutional ambivalence. Their capacity to endure depends on long-term commitment, relational work, and the ability to navigate complex and sometimes conflictual interactions with public authorities. Rather than representing stable end-points, these initiatives should be understood as experimental and fragile configurations, in which transformative ambitions are continuously tested against the realities of Milan’s urban political economy.

The cases discussed in this section show that transformative adaptation among Milan’s NUEs operates through the slow reconfiguration of governance arrangements, infrastructures, and socio-economic relations. While limited in number, these initiatives illuminate the possibilities—and limits—of pursuing structural change from within the city, offering insights into how transformation can be enacted incrementally, collectively, and under conditions of ongoing constraint.

### 4.4 Hybrid and overlapping adaptations: balancing multiple adaptive logics

While the previous sections have discussed coping, incremental, and transformative adaptation as analytically distinct forms, the empirical material shows that several of Milan’s New Urban Economies cannot be adequately understood through a single adaptive lens. Rather than fitting neatly into one category, some initiatives operate in between, holding together different adaptive logics as part of their everyday organisational practice.

A first group of initiatives combines coping and transformative adaptation. In these cases, strong normative orientations and ambitions for systemic change coexist with organisational arrangements that remain fragile, small-scale, or heavily reliant on unpaid work. Solidarity-based initiatives such as the two solidarity purchasing groups (Int_10 and Int_11) articulate far-reaching critiques of dominant food systems and consumption models yet reproduce themselves through forms of collective organisation that depend on voluntary labour and limited institutional support. Their transformative horizon is therefore sustained through coping practices that allow the initiative to endure over time, even as they constrain its capacity to scale or stabilise.

A similar tension characterises initiatives such as the other package-free shop interviewed (Int_1) and the recycling initiative (Int_18), where transformative aspirations are embedded in practices that remain structurally precarious. In the first case, the ambition to promote zero-waste consumption and cultural change encountered persistent economic and bureaucratic pressures, compressing broader transformative goals into everyday efforts to remain viable. The second example, in turn, operates as a cultural and relational practice aimed at redefining norms around reuse and waste, while relying on informal infrastructures and limited resources that require continuous adaptive effort. In both cases, coping and transformation are not sequential phases but simultaneous conditions of action.

Other initiatives occupy an intermediate position between incremental and transformative adaptation. While their organisational models rely on market-compatible tools and professionalised practices, these are mobilised to pursue broader changes in governance, labour relations, or collective awareness. In these cases, activities draw on efficiency, scale, and managerial capacity, yet are coupled with attempts to influence sectoral norms and institutional frameworks (Int_14 and Int_24). Here, incremental innovation does not exhaust the adaptive horizon but serves as a means for pursuing more ambitious forms of change, albeit within the constraints of existing economic arrangements.

Finally, hybrid adaptation also appears in initiatives that combine coping and incremental logics, prioritising stabilisation and qualitative consolidation over growth. This is the case of the already mentioned long-term project of participatory urban regeneration (Int_12), which exemplifies this configuration by sustaining a viable economic activity through careful curation, relational work, and containment of scale. Its adaptive strategy neither seeks expansion nor limits itself to emergency-oriented responses but instead navigates between maintaining economic viability and preserving value coherence.

These hybrid configurations highlight an important empirical insight: adaptation among Milan’s NUEs is rarely a matter of choosing one path over another. Instead, initiatives continuously negotiate between different adaptive logics in response to economic pressure, institutional ambiguity, and the desire to remain faithful to their founding values. Hybridity, in this sense, should not be read as inconsistency or analytical noise, but as a situated and meaningful response to the conditions under which these initiatives operate. This perspective provides a natural bridge to the discussion, which examines how sectoral positioning, temporal trajectories, and policy environments shape the possibilities and limits of adaptive action in Milan’s urban economy.

## 5 Discussion: adaptation as positioning within Milan’s urban economy

The empirical analysis reveals that adaptation among Milan’s New Urban Economies is not a simple choice but a strategic position within a complex urban political economy. The distribution of coping, incremental, and transformative adaptations reflects how different initiatives navigate and are shaped by Milan’s specific configuration of market pressures, policy landscapes, and socio-spatial inequalities (Balducci et al., 2017; Armondi et al., 2024). These patterns also carry important implications for urban governance, as they highlight how policy frameworks and institutional arrangements actively shape the adaptive capacities and trajectories of New Urban Economies.

Incremental adaptation emerges as the dominant configuration, a finding that is structurally intelligible rather than surprising. Milan’s urban environment actively promotes sustainability-oriented innovation, yet often within frameworks that prioritise market compatibility, professionalisation, and growth. Incremental initiatives thrive by translating ethical and ecological values into replicable formats, securing stability and visibility by aligning with prevailing consumer trends and policy incentives (Int_5, Int_9, and Int_16). This does not signify a dilution of ambition but represents a pragmatic mode of inhabiting the urban economy where adaptation is synonymous with strategic alignment. However, this mode largely leaves untouched the structural drivers of inequality and environmental stress embedded in the city’s development model (Feola, 2015).

Coping adaptation, while less prevalent, fulfils a critical and often overlooked function. It represents not passivity but a necessary response to conditions of acute constraint. Coping involves maintaining essential social functions—redistributing food, preserving community purchasing models—under persistent pressures of resource scarcity, volunteer dependence, and institutional ambivalence (Int_3, Int_10 and Int_11). Their value lies in sustaining alternative practices and imaginaries, acting as social buffers, and keeping alive forms of economic democracy and solidarity. Yet, coping can also manifest as a defensive, artisanal resilience in hyper-competitive sectors like fashion, where initiatives survive through restraint and diversification rather than growth (Int_22).

Transformative adaptation, points to a more ambitious mode of engagement (Int_12, Int_15, and Int_8). These initiatives aim to reconfigure the rules of the game itself—whether through participatory governance of urban space, advocacy for labour and mobility justice, or the construction of horizontal agri-food networks. Their adaptation is not about fitting in, nor fixing up, but about altering the socio-material and institutional conditions of urban economic life. Their relative scarcity underscores the significant barriers to pursuing systemic change from within a competitive, financialised urban environment.

Crucially, these adaptive stances are not rigid categories but often coexist in hybrid configurations within single initiatives. Solidarity purchasing groups embody transformative values but rely on the coping mechanisms of volunteer labour. The eco-friendly, bike-based logistics platform (Int_14) combines incremental business efficiency with transformative advocacy. This hybridity is not an analytical shortcoming but a core empirical finding: it reveals adaptation as a continuous, situated negotiation in which initiatives balance survival, innovation, and political ambition across different domains of their operations. This fluidity challenges linear narratives of progression from coping to transformation, highlighting a dynamic process of navigation under polycrisis instead.

Two structural factors powerfully explain this differentiated configuration: sectoral embeddedness and temporal trajectory. The sectoral logic determines the ‘rules of the game’. The food sector, buoyed by policy support like the Milan Urban Food Policy, provides fertile ground for all adaptive types, from coping to transformative. In stark contrast, the fashion sector, characterised by global competition and weak regulation, confines most initiatives to precarious coping or incremental niche branding. The mobility and tourism sectors are characterised by high policy dependence, making transformative adaptation contingent on successful co-design and political advocacy. Thus, an initiative’s transformative potential is heavily mediated by the regulatory and market structures of its sector. This suggests that sector-specific policy environments play a crucial role in enabling or constraining different adaptive pathways.

Simultaneously, a longitudinal perspective reveals that the historical moment of an initiative’s founding leaves a lasting imprint on its adaptive DNA. We observe three generational clusters: the *pioneers* (pre-2010), such as the solidarity purchasing groups, rooted in communitarian and activist traditions; the *crisis responders* (2012–2016), born in the aftermath of the financial crisis and oriented towards pragmatic solutions; and the *urban innovators* (post-2020), native to a Milan where sustainability is a marketised brand (Cucca, 2012). This temporal layering clarifies why incremental adaptation is so prevalent today: Milan’s contemporary political economy attracts and rewards business-oriented sustainability ventures, while the transformative capacity often resides in older, sometimes struggling community structures. Attending to history adds essential depth, underscoring that adaptation is always embedded in a particular spatial–temporal context and conditioned by the urban political economy in which initiatives emerge.

The analysis also surfaces persistent tensions that cut across adaptive types. A central tension is between diversity and convenience. Incremental initiatives often succeed by making sustainability convenient, which risks homogenising alternative practices into marketable formats. Furthermore, many NUEs operate as isolated entrepreneurial efforts, lacking the networks to form a critical mass. This fragmentation limits their collective political agency and bargaining power. Competition remains ambivalent, with similar initiatives being potential allies for networking yet also competitors for a limited market share and consumer attention.

Ultimately, the adaptation of Milan’s NUEs is a process of positioning and negotiation within a rapidly changing and uneven urban context shaped by polycrisis. It is a response to the compounded pressures of economic volatility, spatial inequality, and institutional fragmentation (Istituto Nazionale di Statistica (ISTAT), 2020; Tozzi, 2023). This perspective moves beyond dichotomous readings that frame new entrepreneurial initiatives as either inevitably co-opted or heroically transformative. Instead, it reveals how transformative ambitions are actively sustained, recalibrated, and, in some cases, constrained through the daily work of adaptation (Andres, 2025). The capacity for transformation is not an intrinsic property but emerges from the interplay between an initiative’s values, its sectoral and historical positioning, and its relational engagement with the regulatory and institutional context.

## 6 Conclusion

This article has examined how New Urban Economies in Milan navigate the urban polycrisis through distinct adaptive configurations. By applying Fedele et al.’s (2019) typology, we have reconceptualised adaptation not as a technical adjustment but as a situated, value-laden process of strategic positioning within a competitive and crisis-ridden urban political economy (Balducci et al., 2017; Armondi et al., 2024).

More specifically, our analysis reveals a fragmented situation where incremental adaptation is the predominant mode. This finding reflects the selective integration of sustainability into Milan’s dominant development model. Incremental initiatives innovate within market and policy frameworks, securing viability and visibility while largely reproducing existing socio-economic structures. This challenges celebratory narratives of urban innovation, showing how alternative economies can align with—and even reinforce—growth-oriented trajectories.

At the same time, coping and transformative adaptations remain vital. Coping initiatives provide essential buffers against urban vulnerability, sustaining alternative practices through relational labour and ethical commitment despite institutional neglect. Transformative initiatives demonstrate that structural change is possible, unfolding through the slow, contested reconfiguration of governance, space, and economic relations (Magnaghi, 2020).

Overall, the coexistence and interaction of different adaptive configurations point to the prevalence of hybrid adaptation. Many NUEs combine multiple adaptive logics, highlighting adaptation as a continuous navigation rather than a discrete choice. This hybridity is a pragmatic response to polycrisis, not a sign of incoherence.

These adaptive patterns are shaped by two key structural forces: sectoral embeddedness and temporal trajectory. The food sector, supported by policies like the Milan Urban Food Policy Pact, enables a wider range of experimentation. Furthermore, an initiative’s founding generation imprints its adaptive DNA. Pioneering, activist-rooted projects (pre-2010) prioritise community and autonomy, while newer, post-2020 ventures are often market-native and scalability-oriented. This historical layering explains the current dominance of incremental adaptation.

Taken together, these findings carry significant policy implications. Fostering genuinely transformative urban economies requires moving beyond branding sustainability. It demands structural interventions: ensuring stable, equitable and affordable access to urban space, formally recognising relational and care labour, and developing governance frameworks that support collective and democratic innovation rather than just entrepreneurial ventures. Without such foundations, NUEs will remain constrained, their potential to address root causes of socio-ecological inequality largely unfulfilled.

From a theoretical perspective, this study advances urban political economy and sustainability transitions scholarship by conceptualising adaptation as the primary arena where the politics of urban transformation are negotiated in practice. Rather than viewing transformation as a linear or externally driven process, the findings show that adaptive trajectories are continuously shaped through situated and socio-spatially embedded practices, in which multiple logics coexist and are reworked over time. In this sense, the article contributes to ongoing debates by highlighting the hybrid and negotiated character of urban economic transformation under conditions of polycrisis. In turn, it demonstrates that polycrisis does not merely limit alternative economies, but actively molds their forms, strategies, and horizons of possibility.

At the same time, it is important to acknowledge some limitations that help to clarify the scope and analytical positioning of this study. First, the focus on Milan as a case study does not aim at statistical generalisation, but at developing a contextually grounded understanding of adaptation within a paradigmatic urban setting. As a leading example of neoliberal urbanism, characterised by strong dynamics of globalisation, innovation, and socio-spatial inequality, Milan offers analytically transferable insights that may be relevant for interpreting similar processes in other urban contexts, while remaining attentive to place-specific variations.

Second, the qualitative and multi-method approach adopted in this study prioritises an in-depth exploration of organisational practices, meanings, and situated strategies. Rather than aiming for large-scale comparison, this perspective allows for a nuanced understanding of how adaptation is experienced, negotiated, and enacted within specific socio-spatial contexts.

Finally, the empirical prominence of food-related initiatives within the sample reflects a pattern emerging from the mapping process itself, pointing to the centrality of food as a key domain for the development of New Urban Economies. While this sectoral concentration may shape the observed configurations of adaptation, it also constitutes a relevant empirical finding, suggesting that certain urban domains may offer more fertile conditions for the emergence and consolidation of value-driven economic practices.

Future research should extend this adaptive lens to other urban contexts, investigate municipal policy mechanisms in depth, and analyse the role of digital platforms in reshaping NUE strategies. Ultimately, the central challenge remains: how can cities cultivate environments that enable a shift from prevailing incrementalism toward deeper, more democratic and ecological transformations? The experiences of Milan’s NUEs, in all their constrained diversity, offer critical insights for this urgent task.

## Data Availability

The raw data supporting the conclusions of this article will be made available by the authors without undue reservation.
